# Interaction-Temporal GCN: A Hybrid Deep Framework For Covid-19 Pandemic Analysis

**DOI:** 10.1109/OJEMB.2021.3063890

**Published:** 2021-03-04

**Authors:** Zehua Yu, Xianwei Zheng, Zhulun Yang, Bowen Lu, Xutao Li, Maxian Fu

**Affiliations:** College of EngineeringShantou University 12386 Shantou Guangdong 515063 China; School of Mathematics and Big DataFoshan University 47868 Foshan Guangdong 528000 China; The Second Affiliated Hospital of Shantou University Medical CollegeShantou University 12386 Shantou Guangdong 515063 China

**Keywords:** ARIMA, Covid-19, GCN, time series prediction, traffic forecasting

## Abstract

The Covid-19 pandemic is still spreading around the world and seriously imperils humankind's health. This swift spread has caused the public to panic and look to scientists for answers. Fortunately, these scientists already have a wealth of data—the Covid-19 reports that each country releases, reports with valuable spatial-temporal properties. These data point toward some key actions that humans can take in their fight against Covid-19. Technically, the Covid-19 records can be described as sequences, which represent spatial-temporal linkages among the data elements with graph structure. Therefore, we propose a novel framework, the Interaction-Temporal Graph Convolution Network (IT-GCN), to analyze pandemic data. Specifically, IT-GCN introduces ARIMA into GCN to model the data which originate on nodes in a graph, indicating the severity of the pandemic in different cities. Instead of regular spatial topology, we construct the graph nodes with the vectors via ARIMA parameterization to find out the interaction topology underlying in the pandemic data. Experimental results show that IT-GCN is able to capture the comprehensive interaction-temporal topology and achieve well-performed short-term prediction of the Covid-19 daily infected cases in the United States. Our framework outperforms state-of-art baselines in terms of MAE, RMSE and MAPE. We believe that IT-GCN is a valid and reasonable method to forecast the Covid-19 daily infected cases and other related time-series. Moreover, the prediction can assist in improving containment policies.

## I. Introduction

The outbreak of Covid-19, which began in early 2020, has become a huge threat to human health. As of Jun 28, 2020, statistics from the Johns Hopkins University Center for Systems Science and Engineering [1] show that more than 10 million people have been infected by Covid-19 worldwide. In the United States alone, there are more than 2.5 million cumulative infected cases.

Time-series are the most widely used format of Covid-19 reports. Tendency prediction has become an important pandemic analysis tool because it can inform policies which slow the spread of the pandemic. In Covid-19 records analysis, the tendency features of daily infected cases, the interaction among cities, and the performance of containment policy can be obtained through some technical approaches. Currently, many methods have been applied to Covid-19 records analysis in some countries [2]–[5], but without multi-interactions analysis.

**Fig. 1.**
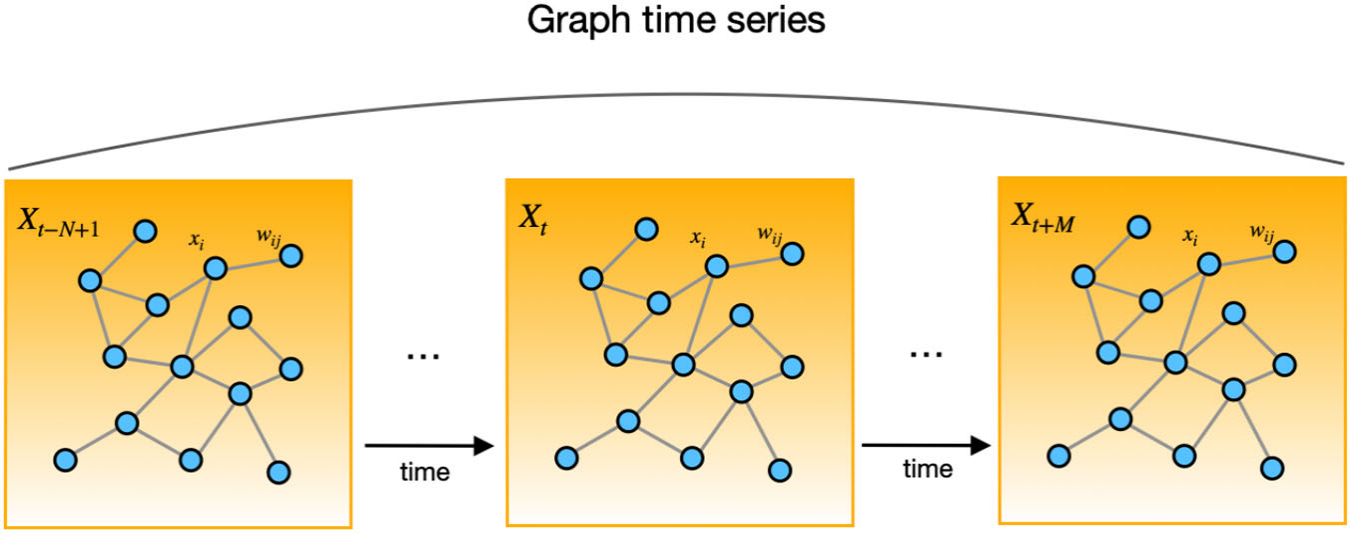
Graph time-series. Each *X_t_* is a frame of current sequence at time step *t*, which can be recorded by a graph time-series data matrix.

As a classical approach, Graph Neural Network (GNN) performs well in spatial-temporal data analysis. By modeling each entity as a node in a graph, GNN captures the multiple complicated relationships among nodes. The current GNN methods are divided into five categories: Graph Recurrent Neural Network (GRNN), Graph Convolution Network (GCN), Graph Autoencoder (GA), Graph Reinforcement Learning (GRL) and Graph Adversarial Methods [6], [15]. GCNs utilize graph convolutions to formulate the variation of data over networks, and thus are suitable for analyzing the Covid-19 records. In short-term forecasting, STGCN [7] based on convolution achieve a high accuracy rate when dealing with the graph sequences, as shown in Fig. 1. The formats of Covid-19 records are diverse and complex, which means they can be classified as spatial-temporal sequence datasets. In this model, a city is represented as a node, and the Covid-19 records of each city are viewed as a sequence on the node. All the nodes are connected by the edges, which represent the linkages among the nodes.

In order to predict the spread of Covid-19, Benvenuto et al. employed ARIMA to forecast the global Covid-19 pandemic [8]. Qin et al. used social media indexes to predict the spreading tendency of Covid-19 [9]. The readers should refer to the literature for further knowledge on Covid-19 record analysis [3], [4], [10]–[14]. The aforementioned works tend to analyze the occurrence of inflection flashpoints rather than focusing on short-term forecasting. Ribeiro et al. employed a variety of popular methods to forecast the Covid-19 outbreak in Brazil [2] and gave corresponding error indicators.

As a classical statistic model, ARIMAs have been widely used. These statistical methods are valid for sampling time-series but they cannot be directly applied to multi-sequences. Therefore, we have introduced the statistical model into GNN to create a hybrid framework for pandemic analyzing and forecasting. Such a framework is able to capture the interaction temporal topology, creating what we call an Interaction-Temporal Graph Convolution Network (IT-GCN).

Effective short-term predictions of daily infected cases based on IT-GCN can provide helpful information to government and professionals. By comparing the prediction results with the ground truth, the efficacy of containment policies can be evaluated. In summary, our main contributions are as follows:

1) A novel framework is proposed which integrates the classical ARIMA into the GCN, in order to create IT-GCN. In this framework, the Covid-19 records are modeled as sequences over a graph and the interactions among cities are captured.
2) Our framework breaks the limitation of the fixed physical spatial topology. We introduce the interaction topology between nodes into the adjacency matrix. It considers many more relationships than fixed physical spatial topology.
3) IT-GCN achieves high accuracy in short-term prediction of Covid-19 daily infected cases in the US. It can provide significant suggestions for containment policies in the Covid-19 pandemic.

## II. Materials and Methods

**Fig. 2.**
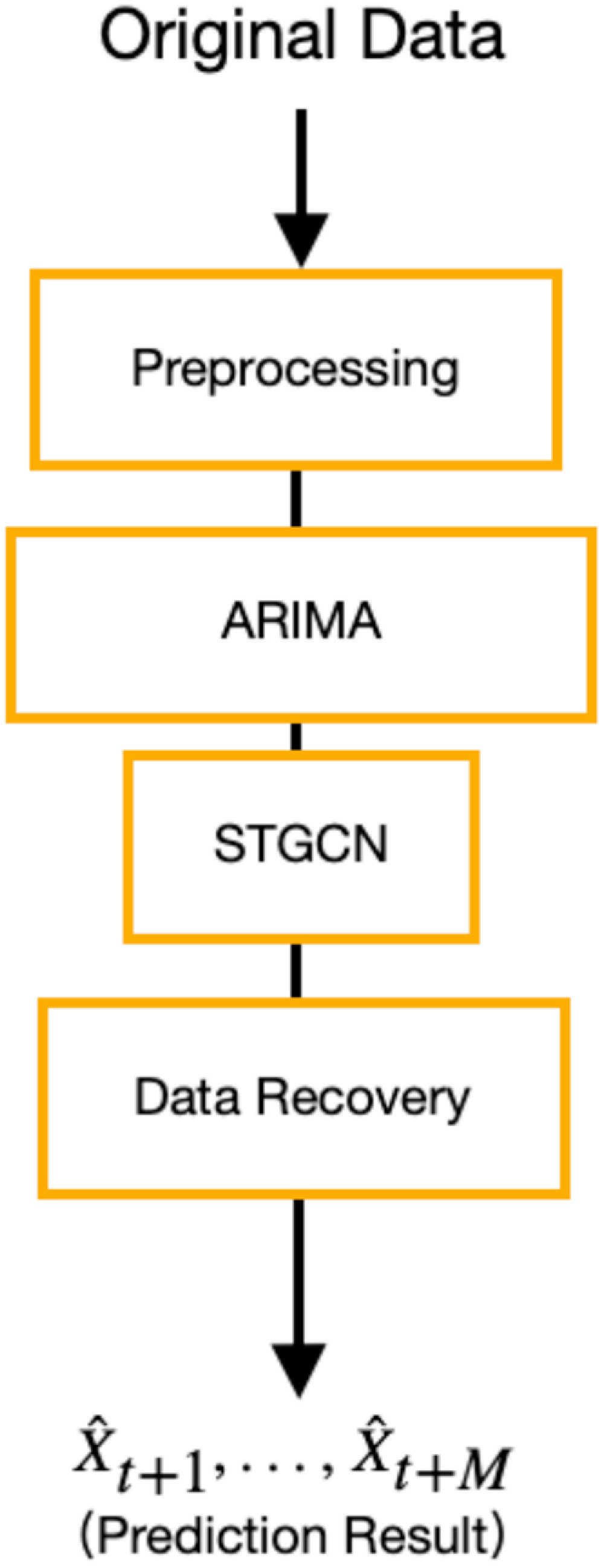
Flow chart of IT-GCN. There are 4 parts (preprocessing, ARIMA, STGCN and data recovery) in IT-GCN.

In this section, we elaborate on the proposed Interaction-Temporal GCN(IT-GCN) illustrated in Fig. 2. The IT-GCN consists of four parts, which are: data preprocessing, ARIMA, STGCN, and data recovery. The details of IT-GCN are described as follows.

### A. Graph Convolution

The convolution of traditional signal processing and CNN [22] cannot be used directly to graph. At present, the integration of graph convolution is divided into two groups [6]: spectral convolution and spatial convolution. We adopt the spectral convolution, which is established by Graph Fourier Transform (GFT). Specifically, the Laplacian matrix of the graph is derived in the spectral domain. We denote "${*_G}$" as the graph convolution operator [16], with the input signal $x \in {R^n}$ and kernel $\theta $,

\begin{equation*}
\theta {*_G}x = \theta \left({Lx} \right): = \theta \left({U{\rm{\Lambda }}{U^T}} \right)x: = U{\rm{\Theta }}{U^T}x\tag{1}
\end{equation*}

where $U \in {R^{n \times n}}$ is the graph Fourier basis, which consists of eigenvectors of the normalized graph Laplacian matrix,

\begin{equation*}
L = {I_n} - {D^{ - \frac{1}{2}}}W{D^{ - \frac{1}{2}}} = U{\rm{\Lambda }}{U^T}\tag{2}
\end{equation*}

where ${I_n}$ is the identity matrix, ${\rm{D}} \in {R^{n \times n}}$ is a diagonal degree matrix, $W \in {R^{n \times n}}$ is the adjacency matrix, ${\rm{\Lambda }} \in {R^{n \times n}}$ is the diagonal matrix of eigenvalues of $L$, and the filter ${\rm{\Theta }}$ is also a diagonal matrix. Under this definition, a graph signal $x$ is filtered by the kernel $\theta $ in (1). In this work, the monitor stations network and the states network are modeled as a weighted undirected graph.

In general, the adjacency matrix ${W_{ij}}$ in GNN is calculated as [17],

\begin{equation*}
{W_{ij}} = \left\{ \begin{array}{ll}
\exp \left({\frac{{d_{ij}^2}}{{{\sigma ^2}}}} \right), & \exp \left({\frac{{d_{ij}^2}}{{{\sigma ^2}}}} \right) \geq 0\\
0, & \text{othervise} \end{array}\right. \tag{3}
\end{equation*}

where $d_{ij}^2$ is the physical distance between nodes $i$ and $j$. However, it only includes the geometric distance between the nodes without considering other interactions. Next, our work aims to break this limitation.

### B. Time-Series Forecasting

To forecast time-series, one could use the historical time-series to estimate the most likely sequence's value at a certain moment in the future, as,

\begin{align*}
&\left({{X_{t + 1}}, \ldots,{X_{t + M}}} \right) = \begin{array}{c} {\arg {\rm{max}}}\\
\scriptstyle{{x_{t + 1}}, \ldots,{x_{t + M}}} \end{array}\\
& P({x_{t + 1}}, \ldots,{x_{t + M}}|{x_{t - N + 1}}, \ldots,{x_t})\tag{4}
\end{align*}

where ${X_t} \in {R^n}$ is a vector aggregate of n nodes in time step $t$.

In this work, we define the network according to states and cities in US to construct a graph and formulate multiple time-series. Especially, we regard the network ${X_t}$ as the nodes of an undirected weighted graph $G$. The graph at each moment is $G({{X_t},W})$ and ${X_t} \in {R^n}$, which has a finite set of nodes, be defined as the characteristic matrix of nodes. $W \in {R^{n \times n}}$ is the adjacency matrix of $G$.

### C. IT-GCN

#### 1) Data Preprocessing

We developed Algorithm 1 to perform the preprocessing. First, it collects and organizes the data into a certain format. After consolidating the original graph time-series $G$, it selects the valid nodes to compose the graph ${G_{selected}}$. Due to the request of ARIMA modeling, the inputs should be stationary. Hence, a stationarity test, the augmented Dickey-Fuller (ADF) test, is adopted to judge whether the time-series meet the requirement of ARIMA modeling or not.

Algorithm 1: **Data Preprocessing.**
**Input:** Original Data
**Output:** Data for experiment Training ${G_{yt}}$, Validation ${G_{yv}}$ and Testing ${G_{ytest}}$;
1: Collecting the initial graph sequences G original from original dataset;
2: Selecting valid nodes, and composing ${G_{selected}}$;
3: Stationarity Testing(ADF test);
4: Calculating m;
5: **return** ${G_{yt}}$, ${G_{yv}}$, ${G_{ytest}}$;

#### 2) ARIMA Progress

Consequently, we used Algorithm 2 to perform IT-GCN. $ARMA({p,q})$ is used to generate the parameters of vector ${{\rm{\Phi }}_i}$ for each time-series, which represent the nodes uniquely in the Euclidean space.

Algorithm 2: **ARIMA Modeling, Forecasting and Data Recovery of IT-GCN.**
**Input:** ${G_y}({{Y_{t - N + 1}}, \ldots,{Y_t}})$
**Output:** Prediction result ${\hat{X}_t}, \ldots,{\hat{X}_{t + M - 1}};$
1: ${G_x}$= m-order difference of ${G_y}$; //Stabilizing processing
2: i=1
3: **while** i <= n **do**
4: t − s = $X_{i,t - n + 1 + m}^m, \ldots,X_{i,t}^m;$
5: ${u_i}$ = ARMA(t − s, order = (p,q));
6: i + +;
7: **end while**
8: //${u_i}$ = $({{\phi _1}, \ldots,{\phi _p},{\theta _1}, \ldots,{\theta _q}})$, ${G_x} = $ $({{X_1}, \ldots,{X_n}})$
9: **for** j = 1; j <= n; j ++ **do**
10: **for** k = 1; k <= n; k ++ do
11: $Dis{t_e}({{X_k},{X_j}}) = {| {| {{\alpha _k} - {\alpha _j}} |} |_2}$;
12: **end for**
13: **end for**
14: **for** i = 1; i <= n × n; i ++ **do**
15: Calculating the adjacency matrix W by (9);
16: **end for**
17: $\hat{G}_x^m = STGCN({G({X,W})});$
18: //Data recovery
19: **while** m > 0 **do**
20: m −−;
21: $\hat{G}_x^{m - 1} = \hat{G}_x^m + G_x^{m - 1}$;
22: **end while**
23: **return** ${\hat{G}_x}$;

The vectors are,

\begin{equation*}
{u_k} = \left({{{\rm{\Phi }}_k},{{\rm{\Theta }}_k}} \right) = \left({{\phi _1}, \ldots,{\phi _p},{\theta _1}, \ldots,{\theta _q}} \right) = \left({{\alpha _1}, \ldots,{\alpha _{p + q}}} \right)\tag{5}
\end{equation*}

where $p$ is the order of model, $\phi $ is the $AR$ parameter and $\theta $ is the $MA$ parameter. Basing on the ${u_k}$ and [18], we define a multivariate equation $f({{u_k}})$ as,

\begin{align*}
&f\left({{u_k}} \right) = \left| {\mathop \sum \limits_{i = 1,j = 1}^{p,q} \left({{\phi _i}{x_{t - i}} + {\theta _j}{\epsilon _{t - j}}} \right) - \left({{x_t} - {\epsilon _t} - C} \right)} \right|\\
& = \left\{ {\begin{array}{l} {\left| {\mathop \sum \limits_{i = 1,j = 1}^{p,q} \left({{\alpha _i}{x_{t - i}} + {\alpha _{j + p}}{\epsilon _{t - j}}} \right) - \left({{x_t} - {\epsilon _t} - C} \right)} \right|, pq \ne 0}\\
{\left| {\mathop \sum \limits_{i = 1}^p {\alpha _i}{x_{t - i}} - \left({{x_t} - C} \right)} \right|,\quad\hspace{62pt} q = 0,p \ne 0}\\
{\left| {\mathop \sum \limits_{j = 1}^q {\alpha _j}{x_{t - j}} + \left({{\epsilon _t} + C} \right)} \right|,\quad\hspace{62pt} p = 0,q \ne 0} \end{array}} \right.\tag{6}
\end{align*}

where, ${x_t}$ is the sequence, $\epsilon $ is noise, and $C$ is constant, which consists the expectation of ${x_t}$. The goodness-of-fit between the ${u_k}$ and the real model of time-series is inversely correlated with the value of $f({{u_k}})$. Then, the equation of ${U_k}$ is,

\begin{equation*}
{U_k} = \mathop {\arg \min }\limits_{{u_k}} \left\{ f\left({{u_k}} \right)|\exists u_k^{\rm{*}}:\mathop {\lim }\limits_{{u_k} \to u_k^{\rm{*}}} f\left({{u_k}} \right) = 0\right\} \tag{7}
\end{equation*}

Ideally, an optimal $u_k^*$ makes $f({u_k^*}) = 0$. However, in practice, we have to find the most approximate value.

In a graph with $n$ nodes, each node has a unique ${U_k}$. The Euclidean distance between any pair of nodes ${u_k}$ and ${u_l}$, $Dis{t_e}({{U_k},{U_l}})$ is given by,

\begin{align*}
\!Dis{t_e}\left({{U_k},{U_l}} \right) \!& =\! \sqrt {{{\left({{\alpha _{k,1}} - {\alpha _{l,1}}} \right)}^2} + \cdots + {{\left({{\alpha _{k,p + q}} - {\alpha _{l,p + q}}} \right)}^2}} \\
&= \sqrt {\mathop \sum \limits_{j = 1}^{p + q} {{\left({{\alpha _{k,j}} - {\alpha _{l,j}}} \right)}^2}}\\
\!& =\! {\left| {\left| {{\alpha _k} - {\alpha _j}} \right|} \right|_2} k,l = 1, 2, \ldots, n\tag{8}
\end{align*}

Then, the adjacency matrix generating equation in IT-GCN is,

\begin{equation*}
{W_{k,l}} = \left\{ \begin{array}{ll}
\exp \left({\frac{{\left| {\left| {{\alpha _k} - {\alpha _l}} \right|} \right|_2^2}}{{{\sigma ^2}}}} \right), & \exp \left({\frac{{\left| {\left| {{\alpha _k} - {\alpha _l}} \right|} \right|_2^2}}{{{\sigma ^2}}}} \right) \geq \epsilon \\
0, & \text{othervise} \end{array}\right.\tag{9}
\end{equation*}

In this work, ${\sigma ^2}$ and $\epsilon $ are thresholds to control the distribution and sparsity of matrix W, which are empirically set as 10 and 0.5, respectively.

#### 3) Forecasting

In STGCN, each spatial-temporal convolutional block is formed as a sandwich structure with two gated sequential convolution layers and one spatial graph convolution layer [7].

#### 4) Data Recovery

We accumulate the original data, ${G_{X_t^{m - 1}}}, \ldots,{G_{X_{t + M - 1}^{m - 1}}}$, and output of STGCN, ${\hat{G}_{X_t^{m - 1}}}, \ldots,{\hat{G}_{X_{t + M - 1}^{m - 1}}}$, to recover the m-order differences to ${\hat{G}_{{X_t}}}, \ldots,{\hat{G}_{{X_{t + M - 1}}}}$. The recovery rules follow,

\begin{equation*}
{\hat{S}_n} = \mathop \sum \limits_{i = 0}^{m - 1} \left({\hat{S}_n^{m - i} + S_n^{m - 1 - i}} \right),\ m > 1\tag{10}
\end{equation*}

and

\begin{equation*}
{\hat{G}_X} = \left\{ \begin{array}{ll}
\left({\hat{S}_0^1 + {S_0}, \ldots,\hat{S}_n^1 + {S_n}} \right),& m > 1\\
\left({{{\hat{S}}_0},{{\hat{S}}_1}, \ldots,{{\hat{S}}_n}} \right), & m > 1
\end{array}\right.\tag{11}
\end{equation*}

where ${S_n}$ is the time-series of node in the graph with $n$ nodes.

The main characteristics summary of our framework are,

1) By modeling the records as a graph and forecasting the graph time-series, IT-GCN is generally valid without a fixed spatial relationship.
2) IT-GCN captures the mathematical dependence and interaction-temporal topology among sequences modelled in graph time-series.

### D. Performance

Traffic data and Covid-19 reports are the processed sequences with multiple interaction topology. Due to the wide use of traffic data in our model test, we used traffic forecasting to verify the performance of our proposed pre-Covid-19 analysis. We performed experiments on two recognized traffic datasets, PeMSD3(Sacramento) and PeMSD7(Los Angeles), collected by California Department of Transportation [19]. The details of each datasets are as below.

**PeMS:** It was collected from Caltrans Performance Measurement System (PeMS) in real-time by over 1200(PeMSD3) and 39000(PeMSD7) sensor stations. We randomly selected 228 stations for our model. The time range in D3 is from March 1 to April 18 in 2020, and the weekdays of May and June in 2012 in D7. The stations recorded an average traffic speed every five minutes. The first 34 days are selected as training data, the rest serve as validation and the test set.

To certify the reasonability and generality of our framework, we utilize it in traffic speed prediction. We follow the setting in the STGCN [7] as the training parameters and use 12 observed points to forecast traffic conditions in the next 15, 30, and 45 minutes (M = 3, 6, 9).

Table I shows the traffic prediction results. Our framework performs better than STGCN, while consuming fewer computing resources. The results indicate that our proposed method of replacing physical distances with interaction topology is effective and reasonable. Furthermore, these results illustrate that IT-GCN can capture the interaction-temporal topology among nodes to achieve accurate forecasting.

**TABLE I:** Performance Comparison of Different Approaches on the Dataset PeMSD7 and PeMSD3

model	PeMSD7(15min/30min/45min)			
	MAPE%	MAE	RMSE	Training epoch
IT-GCN(proposed)	**5.202**/**7.237**/**8.603**	**2.238**/**3.034**/**3.596**	4.102/**5.736**/**6.778**	**40**
STGCN	5.219/7.352/8.822	2.246/3.048/3.605	**4.071**/5.742/6.821	60
ARIMA	12.92/13.94/15.20	5.55/5.86/6.27	9.00/9.13/9.38	–
model	PeMSD3(15min/30min/45min)			
	MAPE%	MAE	RMSE	Training epoch
IT-GCN(proposed)	**4.063** /**4.250** /**4.321**	**2.504**/**2.624**/**2.718**	**3.720**/**3.868**/4.103	**8**
STGCN	4.072/4.277/4.393	2.510/2.656/2.738	3.779/3.869/**3.962**	30
ARIMA	9.33/9.84/10.53	3.85/4.15/4.41	6.04/6.17/6.31	–

## III. Results

Next, we employed the proposed approach to the Covid-19 analysis on a daily confirmed dataset in the United States [1].

### A. Dataset

#### 1) Covid-19 reports in US

This is the data repository for the 2019 Novel Coronavirus Visual Dashboard operated by the Johns Hopkins University Center for Systems Science and Engineering (JHU CSSE) [1]. The folder contains daily time-series summary tables, including confirmed cases, deaths and recovered. All the datasets originate from the daily cases reports. The cumulative number of infected cases is extracted, and the daily infected is the 1-order difference of cumulative cases.

In the pandemic analysis of the United States, we viewed the 51 (including the District of Columbia) states as the 51 nodes in the graph and used the first 70 days as the training set, then the rest served as validation and test set. In the analysis of California, we divided 51 cities of CA into 9 districts, which are denoted as 9 nodes.

### B. Experiment Settings

All experiments were compiled by Python and tested on a Windows10 workstation (CPU: Intel(R) i7-8700 GPU: NVIDIA P1000). We used historical data of 10 days to forecast the rate of change in the daily infected cases in the next 14 days. After outputting the rate of change, the original daily rate of change was used to calculate the daily prediction.

We employed classical ARIMA and Spatial SEIR [21] as the baseline. In Covid-19 pandemic analysis, the model setting of IT-GCN is ARIMA (53,0).

### C. Results

#### 1) Covid-19 Pandemic Prediction in the United States

**Fig. 3.**
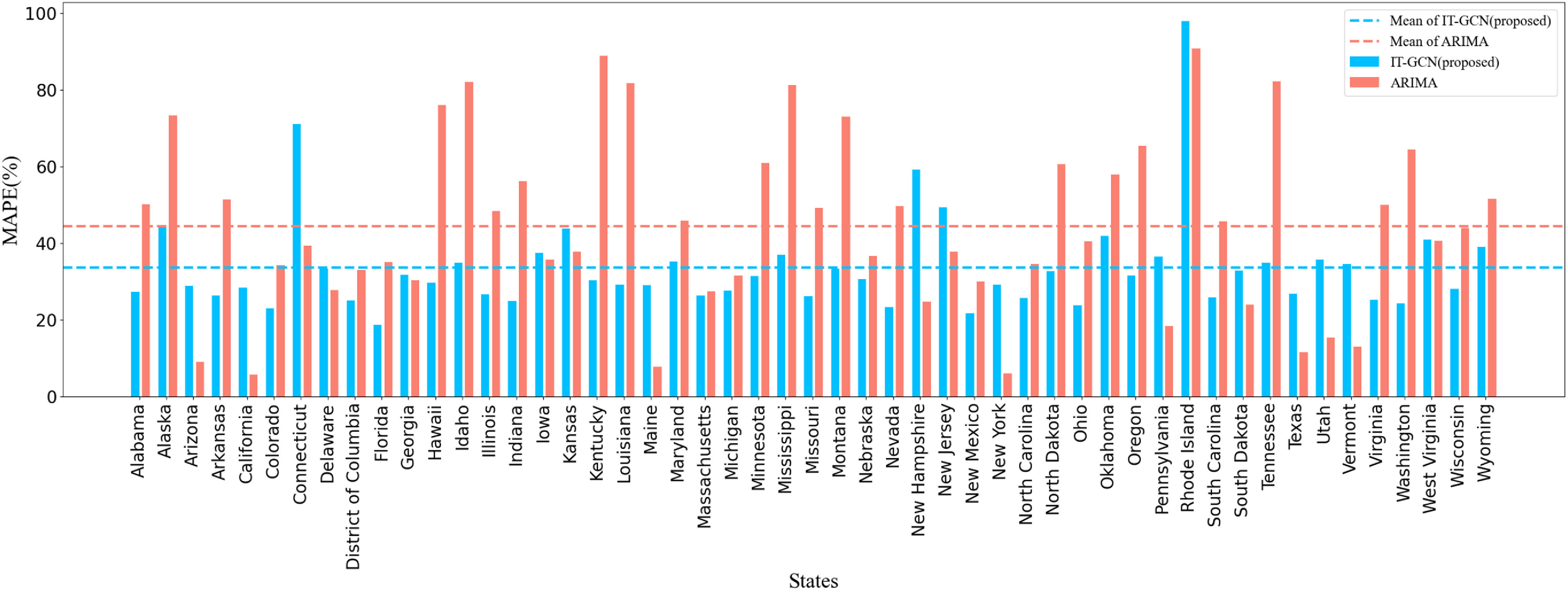
MAPE of Covid-19 daily confirmed infected cases prediction in 51 states of US, 7.11-7.31 2020.

**Fig. 4.**
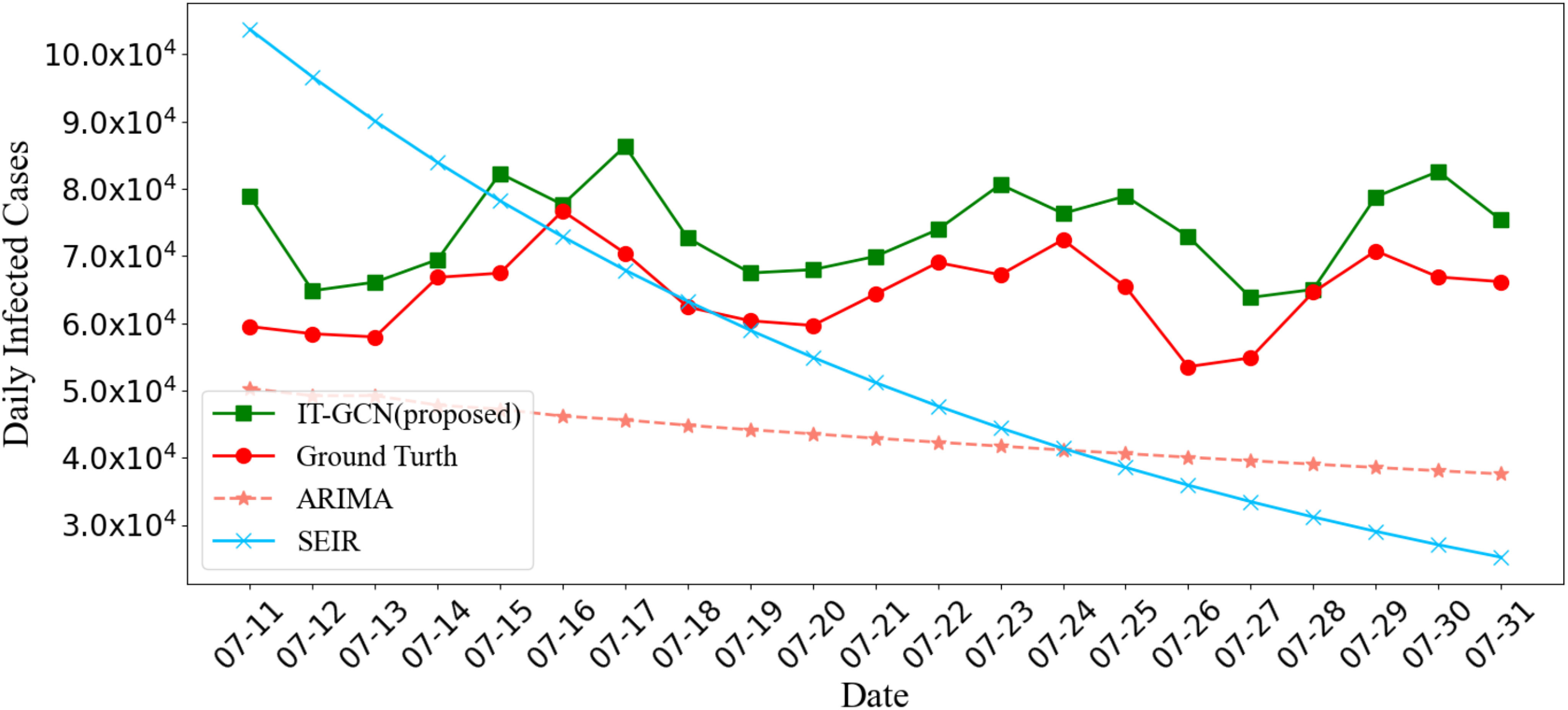
Comparison of Covid-19 daily infected cases prediction in 51 states of US, 7.11-7.31 2020 between IT-GCN (proposed), SEIR and ARIMA [2].

Fig. 3 shows the prediction MAPE of IT-GCN and ARIMA in 51 states during 21-days. The average MAPE of IT-GCN is 33.640%, which is much better than 44.547% of ARIMA. Fig. 4 shows the total prediction results of IT-GCN, SEIR and ARIMA in US, and compares them with the ground truth. We use historical data from March 12th to July 10th to forecast the data from July 11th to July 31st. The records of each 12 days are used to forecast the next outputs of 7 days. Therefore, in Fig. 4, we linked the results of the three prediction parts (7.11-7.17, 7.18-7.24, 7.25-7.31) according to time. The input time lags for each part above are 6.28-7.10, 7.5-7.17 and 7.13-7.24 respectively. In Spatial SEIR, the data from July 10^th^, 2020 are used as the initial data, and all outputs shown in Fig. 4 are prediction results. The results illustrate that IT-GCN performs a lot better than ARIMA and Spatial SEIR compared with the real data in tendency and accuracy. IT-GCN keeps high accuracy in most states and the whole cases. Generally, by learning the features and topology of historical data, IT-GCN forecasts the tendency and increasing of daily infected cases well.

#### 2) Covid-19 Pandemic Prediction in California

**Fig. 5.**
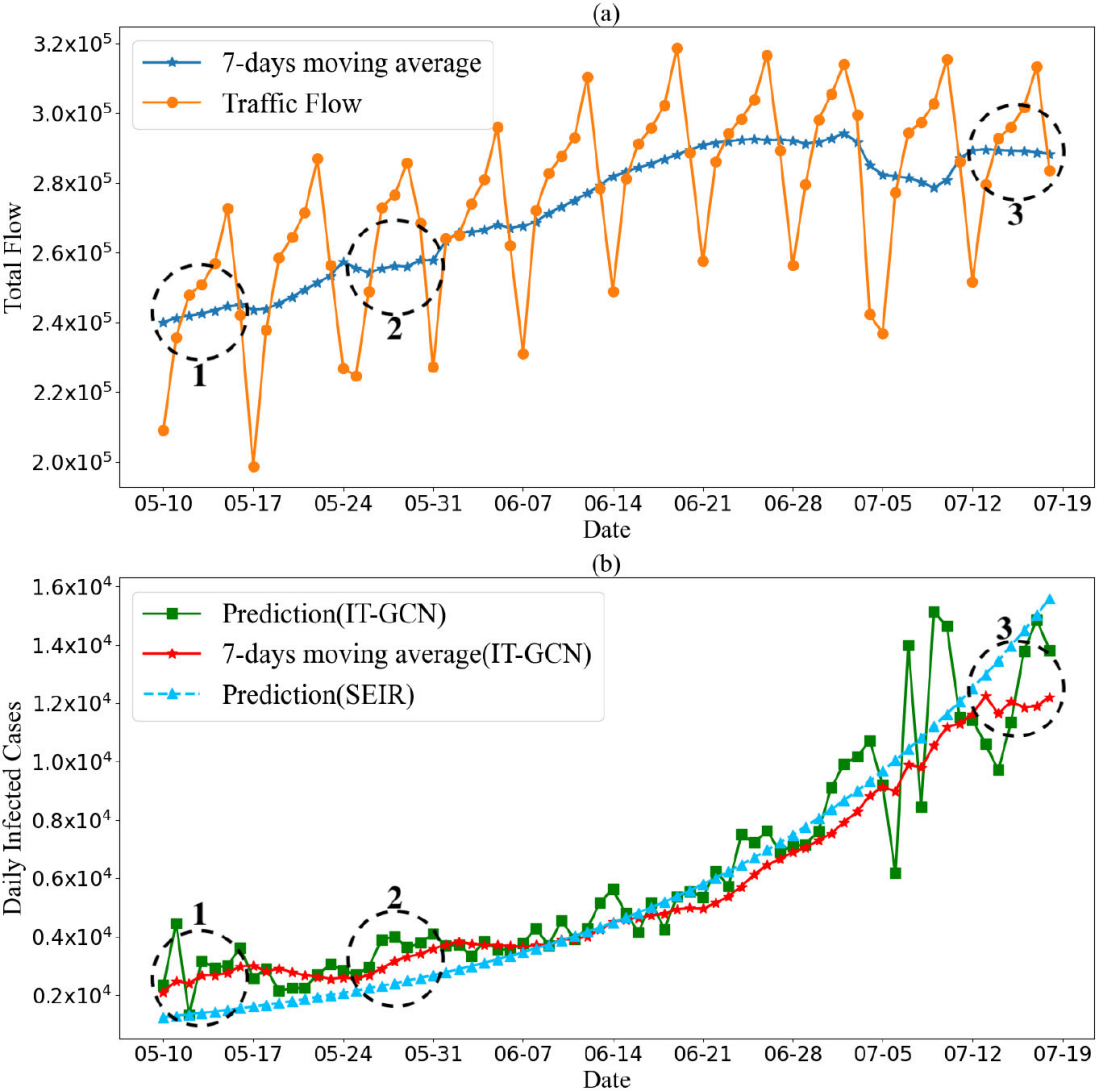
(a): Traffic flow records in California, 5.10-7.19 2020(10 weeks); (b): Covid-19 daily infected cases prediction in California, 5.10-7.19 2020(10 weeks).

Since the pandemic in California is severe and the traffic records in CA are accessible, we chose to focus on the pandemic analysis of CA combined with traffic flow. The results are shown in Fig. 5. From the beginning of May—the period 1 circled in Fig. 5—more and more businesses were allowed to resume work. Fig. 5(a) shows that the traffic flow in this period started increasing weekly. The total flow in weekends was lower than weekdays. Fig. 5(b) shows that the daily infected cases did not increase significantly when businesses reopened. It also illustrates that the containment policies in California were effective in this period.

However, the daily infected cases started increasing in late May. The demonstrations in response to the police killing George Floyd, which occurred in the period 2 circled in Fig. 5, May have directly impacted the outbreak of daily infected cases. In late June, the average traffic flow increased by more than 40000. The prediction results showed that the daily infected cases kept increasing from May to July. At the same time, the traffic flow also kept increasing, which indicated that more and more social activities were held. The high frequency of social activities and the decrease in pandemic containment were two key factors which increased the daily infected cases. These factors led Covid-19 to spread out of control. The government announced the suspension of resumption plan on July 13th, the period 3 circled in Fig. 5. In the period after this announcement, the traffic flow and the prediction of daily infected cases started decreasing, which suggests that the policy worked. According to our prediction and analysis, if containment policies or other containment measures can be deployed earlier, there might be fewer infected cases.

**Fig. 6.**
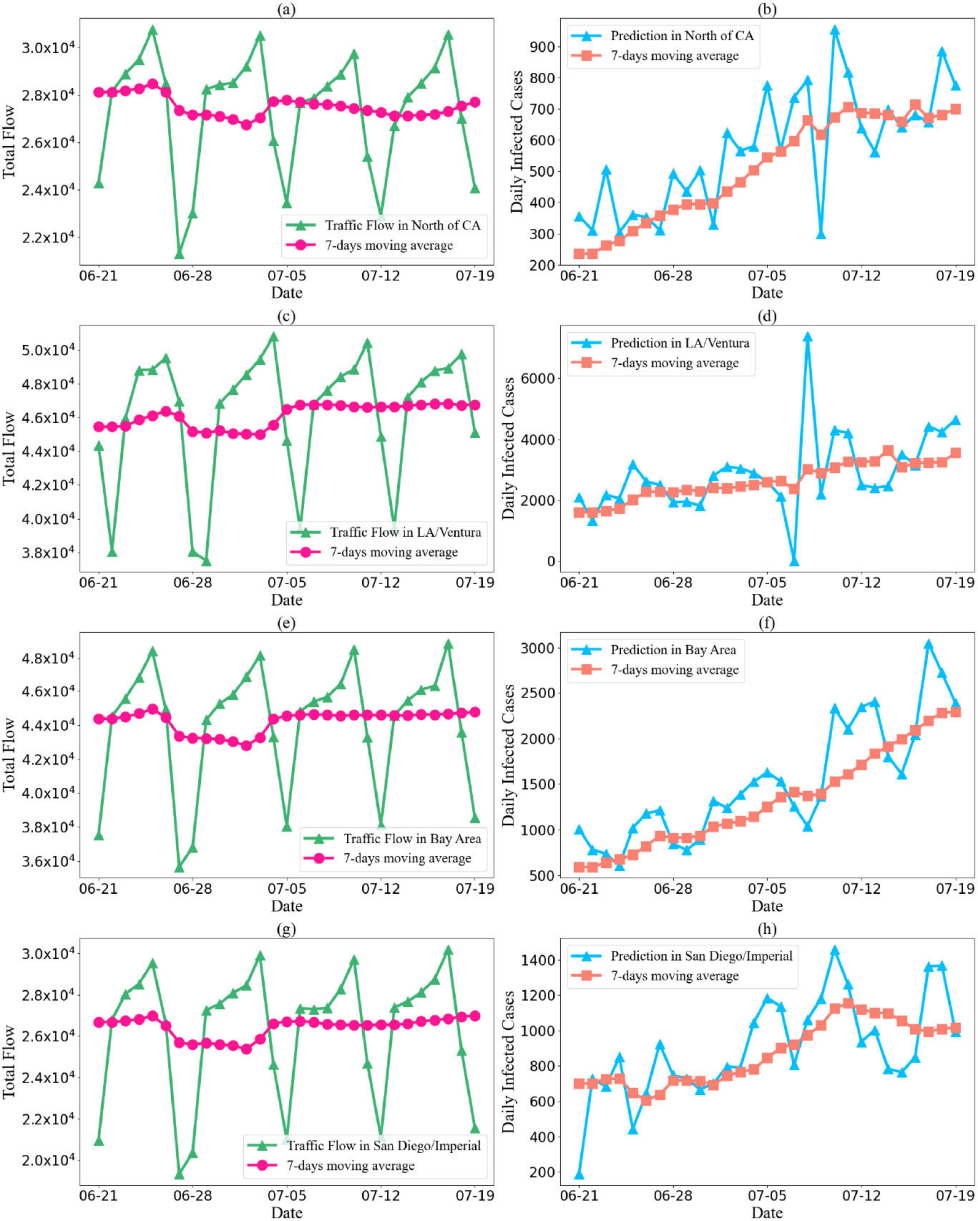
Traffic flow from 6.21 to 7.19 2020(a: North of CA, c: LA/Ventura, e: Bay Area, g: San Diego/Imperia); Covid-19 daily confirmed infected cases prediction from 6.21 to 7.19 2020(b: North of CA, d: LA/Ventura, f: Bay Area, h: San Diego/Imperial).

Fig. 6 shows the traffic flow and Covid-19 predictions in district 3(North(a, b)), district 4(Bay Area(e, f)), district 7(LA/Ventura(c, d) and district 11(San Diego/Imperial(g, h)) in California. By comparing the traffic flow and the prediction of daily infected cases, our algorithm illustrates that the pandemic spreading in most districts was controlled in the middle of July. However, in Bay Area, the cases increased without stopping, which indicated that the situation was still severe. In all, the traffic flow indicated a high crowd density and frequent flow of people, which may be the reason infected cases kept increasing. This discovery is a warning that, in some cities, the containment policies need to be improved urgently.

## IV. Discussion

As the above analysis suggests, the Covid-19 pandemic in California is still widely spreading with limited containment policies. The correlation between traffic density and population densities have been justified in [20], which demonstrates that the increasing of traffic flow will lead the increase of population densities in public and the spreading of Covid-19. The massive traffic flow shows that as people go out more frequently, the daily infected cases keep rising at the same time. Moreover, our results show that the policy effects on pandemic containment can be forecasted and analyzed by IT-GCN. These results also demonstrate that the daily infected cases will decrease with more valid interventions.

Furthermore, to estimate spatial stratified heterogeneity (SSH) of our proposed model, we adopt Q-statistic [22] as a measurement. Evaluated q∈ [01], and the stronger SSH of the inputs, q is closer to 1. The q in Fig 6 is 0.96, and in the three periods in Fig 5, the q is 0.94, 0.93, and 0.94 respectively, which denotes that the diversity among nodes is prominent.

The prediction of daily infected cases is an important reference for containment policies and comparing daily infected cases with traffic flow is helpful. The traffic flow directly represents changes in of social distance and economic activity level. As a result, we can obtain some enlightenments in Covid-19 containment policies development from historical data analysis. By comparing the recent ground truth and predictions, effectiveness of current policies can be appraised. Early understanding of the efficacy of policies positively impacts pandemic containment by avoiding more disruption to the social and public health.

## V. Conclusion

We have developed IT-GCN and used it to analyze the spread of Covid-19. It replaces the spatial distance with the Euclidean distance of the parameter vectors among nodes by an interaction-temporal graph. In Covid-19 analysis, our proposed algorithm provides effective prediction information in daily infected cases in US. Furthermore, these predictions can assist the improvement of containment policies.
